# Copper-Modified Double-Emission Carbon Dots for Rapid Detection of Thiophanate Methyl in Food

**DOI:** 10.3390/foods11213336

**Published:** 2022-10-24

**Authors:** Xiaona Yue, Chunna Zhu, Rongrong Gu, Juan Hu, Yang Xu, Sheng Ye, Jing Zhu

**Affiliations:** College of Science & State Key Laboratory of Tea Biology and Utilization, Anhui Agricultural University, Hefei 230036, China

**Keywords:** carbon quantum dots, copper, thiophanate methyl, rapid detection

## Abstract

The detection of food safety and quality is very significant throughout the food supply. Stable dual-emission copper-modified fluorescent carbon dots (Cu-CDs) were successfully synthesized by a simple and environment-friendly hydrothermal, which was used for the real-time detection of pesticide residues in agricultural products. By optimizing the reaction conditions, Cu-CDs showed two emission peaks, with the highest fluorescence intensities at 375 and 450 nm. The structure, chemical composition and optical properties of Cu-CDs were investigated by XRD, TEM and IR. The results showed that thiophanate methyl (TM) could induce fluorescence quenching of Cu-CDs with no other ligands by the electron transfer through π-π stacking. The synchronous response of the dual-emission sensor enhanced the specificity of TM, which showed remarkable anti-interference capability. The fluorescence quenching degree of Cu-CDs had a good linear relationship with the TM concentration; the low detection limit for a pear was 0.75 μM, and for an apple, 0.78 μM. The recoveries in the fruit samples were 79.70–91.15% and 81.20–93.55%, respectively, and the relative standard deviations (RSDs) were less than 4.23% for the pear and less than 3.78% for the apple. Thus, our results indicate the feasibility and reliability of our methods in detecting pesticide residues in agricultural products.

## 1. Introduction

Food safety has been a widely concerned global human health problem. It is of great significance to synthesize new materials that can quickly detect pesticide residues in agricultural products. Carbon dots (CDs) are defined as a set of sp^2^/sp^3^/sp^2^-sp^3^ hybrid carbon entities with a very small size of about 10 nm, which have specific quantum constraints, edge effects and defects [1,2,3,4]. The emergence of CDs has brought new opportunities and challenges to different scientific fields, mainly due to the adjustable band gap and unique photoelectric properties, namely photoluminescence [5,6,7]. CDs also have excellent biocompatibility, good chemical stability and excellent electron transfer effect, which give unique fluorescent features and functions compared with the traditional inorganic semiconductor quantum dots and organic dyes [8,9,10,11,12]. Thus, CDs are widely used in biological imaging, molecular detection, photoelectric devices, solar cells and other fields [13,14,15,16,17]. Although many scholars have synthesized CDs, a sustainable synthesis and purification process remains a challenge; thus, we have developed a facile synthetic method to allow CDs to receive more extensive applications and replace metal quantum dots, which are toxic to the environment and humans [18,19,20,21]. Compared with the commonly used nonmetallic modification methods, changing the electronic structure of CDs to provide more active sites is easier, for they have more electrons and vacant orbitals. By introducing transition metal modification, the electronic structure and optical properties of CDs can be adjusted, which greatly expands the application of CDs in catalysis, electricity conduction and so on [22,23,24]. Although transition metals with modified CDs show fluorescent stability, such as cadmium (Cd), tellurium (Te), silver (Ag), selenium (Se) and cobalt (Co), they have low biological compatibility that has limited their application [25]. Additionally, the preparation of these materials is toxic and incurs an expensive cost to a great extent, which restricts their application in the fields of biology, medicine and so on [26,27,28,29]. Therefore, it is necessary to develop metal-modified CDs with low cytotoxicity. Among them, copper is used to modify CDs due to its negligible toxicity and easy interaction of functional groups onto the surface of CDs [30].

In order to control fungus and ensure the high quality and high yield output of agricultural products, pesticides are widely used in agricultural production activity [31]. In fruit and vegetable fields, TM is often used to prevent and control various diseases in crops, as well as for postharvest food storage and preseed planting treatment [32,33]. Studies have shown that long-term exposure to TM can pose high risks to human health due to its potential genotoxicity, neurotoxicity, reproductive toxicity and teratogenicity [34,35,36]. The maximum residue limit standard for TM in fruit is 6 mg/kg, as determined by the EU [37]. Thus far, there are many methods to detect TM. The routine food quality and safety analytical methods are gas chromatography (GC), high-performance liquid chromatography (HPLC), mass spectrometry (MS) and capillary electrophoresis (CE) [38,39,40,41], but they all have the disadvantages of expensive instrument cost and time-consuming operation. Therefore, it is urgent to build an efficient and fast TM-detection method.

**Figure 1:**
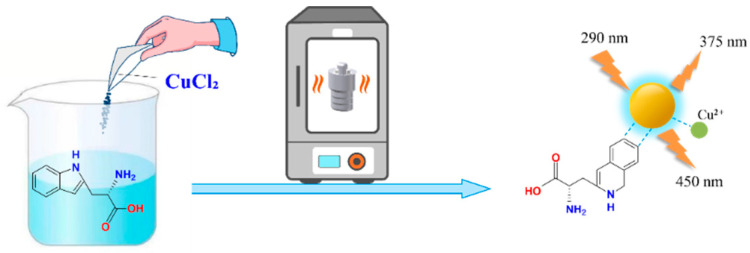
Synthesis route of Cu-CDs.

**Figure 2:**
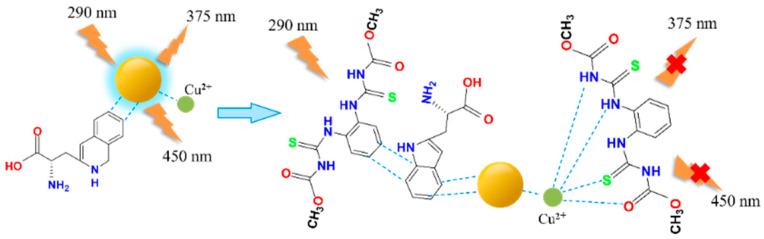
TM detection using Cu-CDs.

We introduced Cu^2+^ onto CDs to synthesize double-emission fluorescence quantum dots (Figure 1). The synthesis process has the advantages of a simple operation, cheap and easy access to raw materials and environmental protection. N, S and O elements with lone pair electrons in TM can chelate with Cu^2+^, and benzene ring in TM can occur in π-π recognition with a benzene ring in Cu-CDs, which leads to fluorescence quenching [4]. Therefore, a synchronous bipeak response strategy for the detection of TM was established in our work (Figure 2). We also provided fast, accurate and reliable information for the quantitative determination of TM in practical samples.

Ligand-free copper quantum dots are synthesized in facile steps; the Cu-CDs in our study can provide a rapid and sensitive detection method for TM residues in agricultural products. In addition, owing to their good stability and a high recovery rate, Cu-CDs have broad application prospects in the field of food safety analysis.

## 2. Materials and Methods

### 2.1. Materials

Potassium chloride (KCl), silver nitrate (AgNO_3_), potassium dihydrogen phosphate (KH_2_PO_3_) and disodium hydrogen phosphate (NaH_2_PO_3_) were purchased from Sinopharm Group Chemical Reagent Co., Ltd., Shanghai, China. Copper dichloride dihydrate (CuCl_2_ · 2H_2_O) and cobalt nitrate (Co(NO_3_)_2_) were purchased from Tianjin Damao chemical reagent factory, Tianjin, China. Sodium chloride (KCl), cadmium chloride (CdCl_2_), chromium trichloride (CrCl_3_), calcium sulfate (CaSO_4_) and aluminum nitrate (Al(NO_3_)_3_) were purchased from Xilong Chemical, Shantou, China. Zinc nitrate hexahydrate (Zn(NO_3_)_2_ · 6H_2_O) and magnesiumchloridehexahydrate (MgCl_2_) were purchased from Jiuding Chemical Technology Co., Ltd., Shanghai, China. Monocrotophos, chlorpyrifos and methyl parathion were obtained from Beijing Boao Yisi Biotechnology Co., Ltd., Beijing, China. L-tryptophan, propyl bromophosphorus, glufosinate-ammonium, glyphosate, chlorpyrifos methyl and thiophanate methyl were acquired from Yuanye Biotechnology Co., Ltd., Wuhan, China.

### 2.2. Apparatus

Transmission electron microscope (TEM) images were recorded using a model S-4800 (Hitachi, Hitachi, Japan). The absorption spectra were performed on a TU-1901 spectrophotometer (puxi General Instrument, Beijing), and the wavelength range was 200–600 nm. The infrared spectrum was measured by a NICOLET IS10 Fourier transform infrared spectrometer (Thermo Fisher, MA, America), and the samples were pressed with KBr. The fluorescence spectrum was measured on a fluorescence spectrometer, model G9800A (Agilent Technologies Inc, Palo Alto, CA, America). The energy spectrometer and X-ray photoelectron spectrum were conducted in a Thermoscientific K-Alpa+ (Thermofisher) where the diffraction angle range was 2θ = 10–80°, and the type of XRD sample holder was a glass, square groove (20 × 10 × 0.5). XRD was carried out at a scanning speed of 4°/min. The voltage and current of the X-ray tube were 15–60 kV, 1 kV/Step, and 6–80 mA, 1 mA/Step, respectively. The type of optical path was focusing light.

### 2.3. Synthesis of Copper-Modified Double-Emission Carbon Dots

L-tryptophan and CuCl_2_ · 2H_2_O were dissolved in 20 mL of H_2_O at a ratio of 1:1.7. After 5 min of ultrasonic cleaning, a mixed clear-blue solution was obtained. The solution was poured into a specific 50 mL reaction container and placed in a constant temperature oven at 180 ℃ for 6 h, then centrifuged at 10,000 rpm for 12 min. Finally, a 0.22 μm microporous filter was used to filter the supernatant in order to obtain a relatively purified product. It was stored in a refrigerator at 4 °C for future experimental use.

The frequency of the ultrasonic cleaner (SB-5200DT, Ningbo Xinzhi Biotechnology Co., Ltd., Ningbo, China) was 40 kHz. An electro-thermostatic blast oven (DHG-91010A, Shanghai Sanfa Scientific Instrument Co., Ltd., Shanghai, China) had a temperature fluctuation and uniformity of ±0.5 °C. A desk centrifuge (TG16-WS, Hunan Xiangyi Co., Ltd., Hunan, China) had a maximum speed of 16,000 rpm and a maximum capacity of 6 × 50 mL. The Cu-CDs samples could be stored at 4 °C for about one month in a refrigerator (BCD-223WDPT, Haier, Qingda, China).

### 2.4. Fluorescence Analysis and Detection of TM by Copper-Modified Double-Emission Carbon Dots

The dual-emission ligand-free Cu-CDs synthesized by the hydrothermal method could be used to detect TM directly. Therefore, the interaction between Cu-CDs and TM and other pesticides and metal ions in a buffer solution was studied. Their quenching phenomenon was monitored by a fluorescence spectrometer. Exactly 20 μL of diluted Cu-CDs solution was added to 4.28 mL of PBS buffer solution (pH = 7.5, 10 mmol/L). Metal ion solutions (K^+^, Na^+^, Mg^2+^, Zn^2+^, Al^3+^, Cr^3+^, Cd^2+^, Ag+ and Co^2+^) and some organophosphorus pesticides (monocrotophos, chlorpyrifos, profenofos, glufosinate, glyphosate, chlorpyrifos methyl, parathion methyl and TM) were added to the Cu-CDs solution, with the final volume of the solution totaling 4.5 mL. Then, they were incubated at room temperature for 2 min to determine the fluorescence emission spectrum of the solution. All fluorescence measurement samples were made in triplicate for further statistics. When the reaction time was 5 min, fluorescence quenching could be observed, but when the reaction time was extended to 30 min, the fluorescence intensity did not change.

### 2.5. Sensitivity Detection of Copper-Modified Double-Emission Carbon Dots to TM

In order to study the applicability of Cu-CDs, we diluted the Cu-CDs solution six times when exposed to different concentrations of the TM solution, then it was diluted with PBS buffer to 4.5 mL and measured the fluorescence quenching spectrum of the solution. Then, we calculated the detection limit of TM using the formula LOD = 3SD/KSV, where SD is the standard deviation of fluorescence intensity when the blank solution (i.e., without TM) is added, and KSV is the slope of the linear fluorescence quenching diagram. All samples with fluorescence measurements were repeated three times for further statistics.

### 2.6. Applicability Experiment of Copper-Modified Double-Emission Carbon Dots

In order to explore the potential applicability value of the prepared sensor in practical application, pears and apples were selected as the objects of analysis and research to detect the residues of TM. The preparation of the sample solution was slightly modified from the previously described method [42]. Then, a 25 mL methanol solution was added, and an ultrasound was performed for 10 min to make the solution fully and uniformly dissolved. The sample solution was centrifuged at 10,000 rpm for 8 min to remove insoluble impurities and then filtered with a 0.22 μm filter membrane. Finally, different concentrations of TM were added to the sample solution, and the solution was analyzed and detected by a fluorescence analyzer, which was repeated three times.

## 3. Results and Discussion

### 3.1. Reaction Conditions Optimization of Copper-Modified Double-Emission Carbon Dots

#### 3.1.1. Proportion of Reactants

When an appropriate amount of copper ion is added to the reaction system, the excited electrons can be transferred to the corresponding copper level. Therefore, the ratio of raw materials in the reaction system was optimized, and the results are shown in Figure S1A. When the ratio of L-tryptophan to copper chloride was 1:0, there was no obvious double emission peak. When the ratio was 1:1.7, 1:2.7 and 1:3.7, the double emission peak appeared, and the intensity of the double emission fluorescence peak decreased with the increase in copper chloride content, which may be attributed to the blockage of surface defects caused by excessive copper, resulting in decreased fluorescence intensity. When the ratio was 1:1.7, the intensity of the two emission peaks was the strongest, which is conducive to the application of Cu-CDs in analysis. Therefore, 1:1.7 was selected as the best ratio for the preparation of Cu-CDs.

#### 3.1.2. Temperature of Reactants

In order to obtain good optical performance, the temperature was also optimized (Figure S1B). The experimental results showed that, with the continuous increase in temperature, the intensity of the prepared Cu-CDs double emission peak also increased. At 180 °C, the double emission peak of Cu-CDs was the most obvious, and the fluorescence intensity was the strongest. When the temperature was higher than 180 °C, the double emission peak intensity gradually decreased, likely due to the high temperature destroying the surface state of carbon dots. Therefore, 180 °C was selected as the best synthesis temperature for subsequent experiments. 

#### 3.1.3. Time of Reactants

Figure S1C shows the effect of time on the fluorescence emission intensity of the prepared Cu-CDs. When the reaction time was prolonged, it was seen that the position of the Cu-CDs double emission peak was increased, and the double peak characteristic was most obvious at 6 h. In order to obtain a Cu-CDs solution with obvious bimodal and strong fluorescence intensity, the optimal reaction time was 6 h.

### 3.2. Related Characterization of the Structure

**Figure 3:**
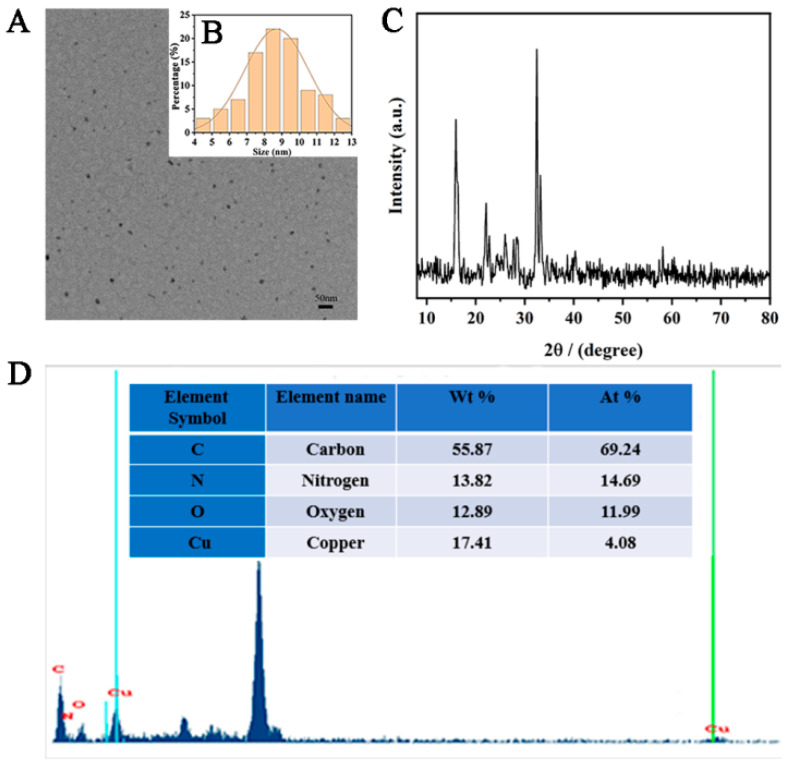
(**A**)TEM image, (**B**) particle size distribution, (**C**) X-ray diffraction and (**D**) EDS of Cu-CDs.

A few brown-yellow powdered Cu-CDs were dissolved in ethanol and placed on a copper net. The morphology and structure of the synthesized products were characterized by transmission electron microscopy (TEM). The Cu-CDs showed a uniform and regular spherical structure with very good dispersion and no obvious agglomeration phenomenon (Figure 3A). The size distribution showed that our Cu-CDs had an average diameter of about 8.89 nm (Figure 3B). We also investigated the crystal structure of the prepared Cu-CDs at room temperature. As shown in Figure 3C, the XRD patterns of Cu-CDs in the diffraction angle range was 2θ = 10–80°. The diffraction peaked at 15.55° and 22.26° and showed that the prepared Cu-CDs had an amorphous structure and no complete or continuous crystals [43]. The diffraction peaks at 27.73°, 33.11° and 32.50° were the (110) planes of copper compounds including Cu_2_O and CuO. The diffraction peak at 40.35° indicated the existence of copper coordination or a doped structure in the synthesized carbon point [43]. Figure 3D shows the EDX diagram of Cu-CDs to study the elemental composition and content of the prepared Cu-CDs. As shown, the Cu-CDs were composed of C, N, O and Cu elements. The atomic weight and mass percentages of C, N, O and Cu were 55.87, 13.82, 12.89, and 17.41 and 69.24%, 14.69%, 11.99%, and 4.08%, respectively.

**Figure 4:**
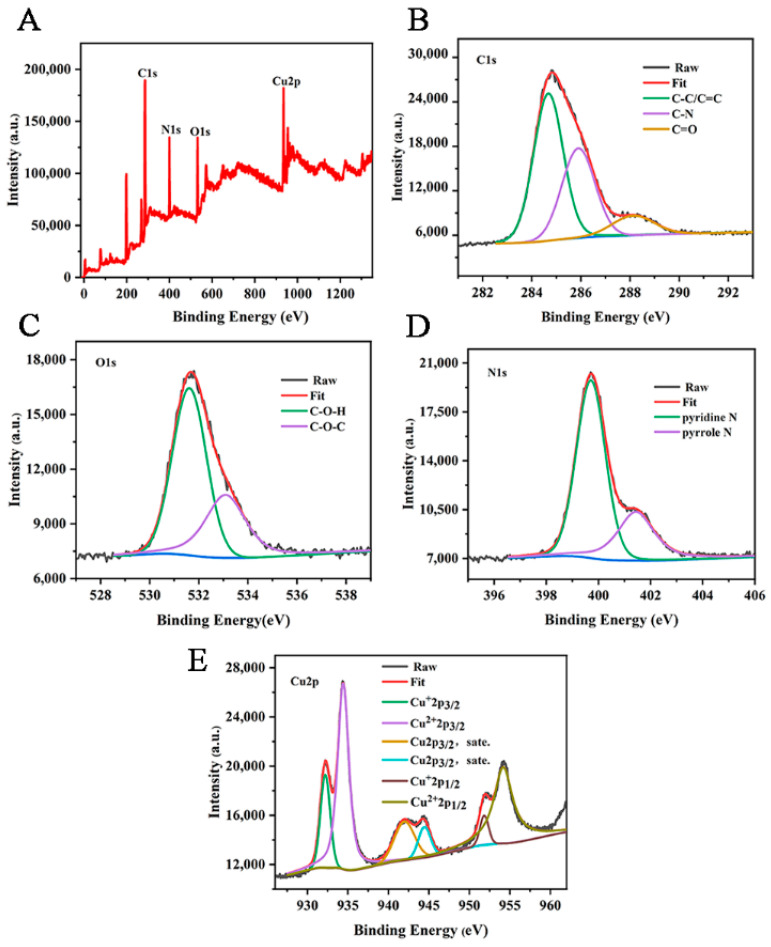
(**A**) XPS full-spectrum scan and high-resolution spectra of C 1s (**B**), O 1s (**C**), N 1s (**D**) and Cu 2p (**E**) of Cu-CDs.

The chemical elements and valence composition of Cu-CDs were further determined by XPS. As can be seen from Figure 4A, the XPS spectrum of Cu-CDs showed four characteristic peaks at 284.98 eV, 531.80 eV, 399.70 eV and 954.25 eV, corresponding to C 1s, O 1s, N 1s and Cu 2p, respectively. A high-resolution spectrum of C1s (Figure 4B) showed three main peaks at 284.67 eV, 285.89 eV and 288.12 eV, which we attributed to C-C/C=C, C-N and C=O groups, respectively. In the high-resolution spectrum of O1 s (Figure 4C), the peaks at 531.61 eV and 533.08 eV corresponded to C-O-H and C-O-C. The high-resolution spectrum of N1s (Figure 4D) showed double peaks of 399.9 eV and 401.7 eV, indicating the presence of pyridine N and pyrrole N [44]. Cu 2p high-resolution spectroscopy (Figure 4E) showed six peaks located at 932.20 eV, 934.42 eV, 941.97 eV, 944.45 eV, 951.82 eV and 954.18 eV. Satellite peaks Cu^+^ 2p^3/2^, Cu^2+^ 2p^3/2^, Cu 2p^3/2^ and Cu^+^ 2p^1/2^, Cu^2+^ 2p^1/2^ represent C-Cu-N and O-Cu bonds, respectively [45]. These analyses show that copper atoms were successfully incorporated into Cu-CDs.

**Figure 5:**
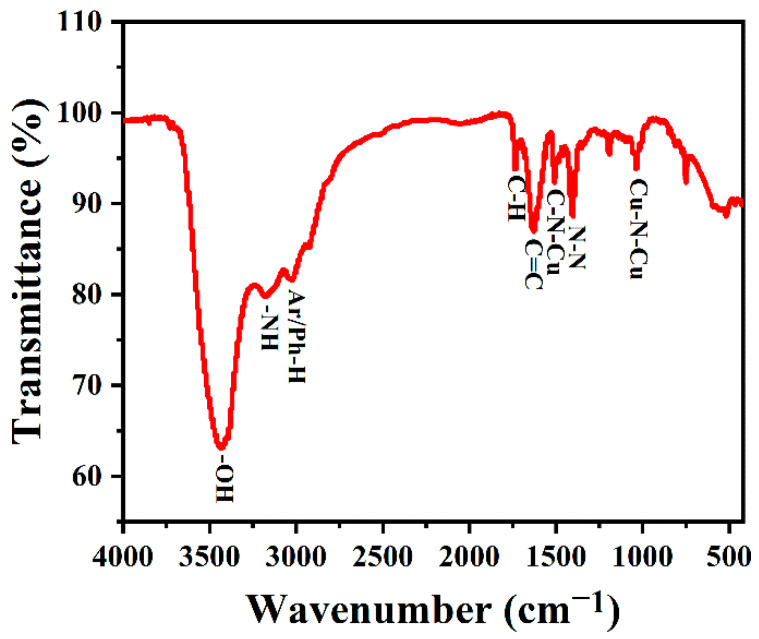
FTIR spectra of Cu-CDs.

TQ Analyst software was used for spectral analysis. After adding KBr, the samples were made into tablets for infrared testing. The FTIR spectra were used to further study the composition of the surface functional groups of the synthesized copper-doped quantum dots. As shown in Figure 5, the characteristic peaks at 3450 and 3170 cm^−1^ belong to O-H/N-H stretching vibration, and the C-H stretching vibration of the indole ring of l-tryptophan is about 2920 cm^−1^. Meanwhile, the band at 1735 cm^−1^ was attributed to the frequency doubling of hydrocarbons outside of the benzene ring, which reflects the existence of its conjugate plane. The absorption peak at 1629 cm^−1^ belongs to the C=C stretching vibration of an aromatic ring, and the absorption peak at 1403 cm^−1^ belongs to N-N stretching vibration, where the bands near 1509 and 1035 cm^−1^ are C-N-Cu and N-Cu-N stretching vibrations, respectively [46], which proved that copper was successfully incorporated into the carbon quantum dots.

### 3.3. Related Characterization of Optical Properties

**Figure 6:**
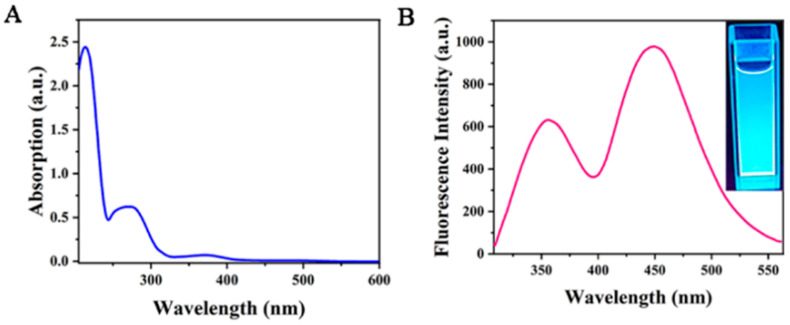
UV–visible absorption spectra (**A**) and fluorescence emission spectra (**B**) of Cu-CDs.

In order to further explore the structure and optical properties of Cu-doped double-emission carbon quantum dots (Cu-CDs), their UV–Vis absorption was measured (Figure 6A). From the figure, we can observe that the absorption peak is centered at 220 nm and corresponds to the π→π * transition of the aromatic ring sp2 domain. The other peak appeared at 286 nm with strong emission intensity, which was caused by the surface state capturing the excited-state energy of Cu-CDs. The illustration in the upper right shows an image under ultraviolet light. Figure 6B shows that Cu-CDs had two obvious fluorescence emission peaks at 375 nm and 450 nm under the excitation wavelength of 290 nm. The emission peak at 375 nm is attributed to the fluorescence of the carbon nucleus, and the emission peak at 450 nm is attributed to the fluorescence caused by the d-d transition of Cu^2+^ [29]. 

### 3.4. Optimization of Copper-Doped Double-Emission Carbon Dots Detection Performance 

The acidity and alkalinity of the reaction system will have a great influence on the emission of double emission peaks, so we designed a series of different pH schemes to obtain the most appropriate pH. As shown in Figure S2A, when the pH value of the reaction system was 3.12–7.5, the intensity of the double emission peak of Cu-CDs increased with the increase of the pH value. When the pH value of the reaction system was 9.08–11.16, the peak of Cu-CDs at 450 nm disappeared with the increase in the pH value. Under acidic conditions, the low pH limited the surface state of Cu-CDs and reduced the fluorescence intensity of the carbon core to 375 nm. A reaction system with high alkalinity can easily destroy the structure of the carbon point and TM. When the pH value was 7.5, the fluorescence emission of Cu-CDs peak intensity reached its maximum. A PBS buffer solution with a pH of 7.5 was selected as the follow-up experiment.

We also investigated the influence of temperature on the fluorescence intensity of Cu-CDs. As shown in Figure S2B, the temperature within the range of 10–75 °C had almost no obvious influence on the fluorescence intensity of the dual emission peak of the Cu-CDs sensor. In addition, the fluorescence intensity of Cu-CDs in the presence of thiophanate methyl (200 μM) at different reaction intervals was investigated, as shown in Figure S2C. The fluorescence quenching phenomenon could be observed when the reaction time was 5 min, and the fluorescence intensity did not change when the reaction time was extended to 30 min. This indicates that Cu-CDs sensors can be quickly used for the detection of TM.

### 3.5. Selective Detection of Copper-Doped Double-Emission Carbon Dots

**Figure 7:**
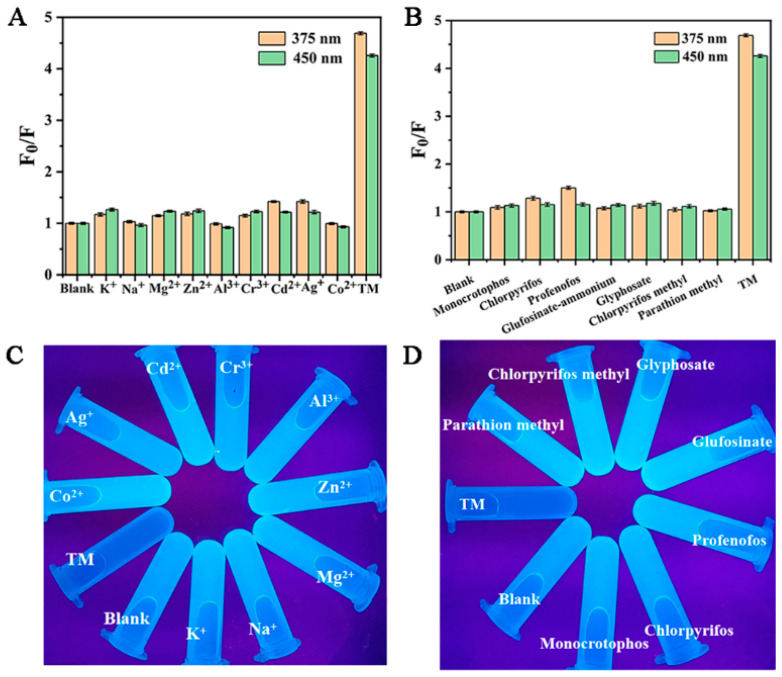
Cu-CDs selectivity to different metal ions (**A**) and to pesticides (**B**), fluorescence images of Cu-CDs solutions with different metal ions (**C**) and Cu-CDs solutions with different pesticides under UV lamp (**D**).

For an excellent dual-emission probe, high selectivity is a basic method to study the detection of thiophanate methyl by Cu-CDs. Under optimized experimental conditions, the addition of other common metal ions, such as K^+^, Na^+^, Mg^2+^, Zn^2+^, Al^3+^, Cr^3+^, Cd^2+^, Ag^+^ and Co^2+^, and organophosphorus pesticides, such as monocrotophos, chlorpyrifos, profenofos glufosinate, glyphosate, chlorpyrifos methyl, parathion methyl and TM in Cu-CDs, was studied. As shown in Figure 7A,B, these results indicated that the fluorescence quenching degree of TM on Cu-CDs was much higher than that of other metal ions and pesticides. Additionally, Figure 7C,D show the fluorescence colors of Cu-CDs, the mixed solutions of different metal ions and Cu-CDs, and the mixed solutions of different pesticides. It can be seen that TM could cause the blue fluorescence quenching of Cu-CDs, while the fluorescence brightness of Cu-CDs hardly changed in the presence of other ions and pesticides. In conclusion, the prepared Cu-CDs have excellent selection ability for TM detection.

### 3.6. Detection of Sensitivity of Copper-Modified Double-Emission Carbon Dots to TM

**Figure 8:**
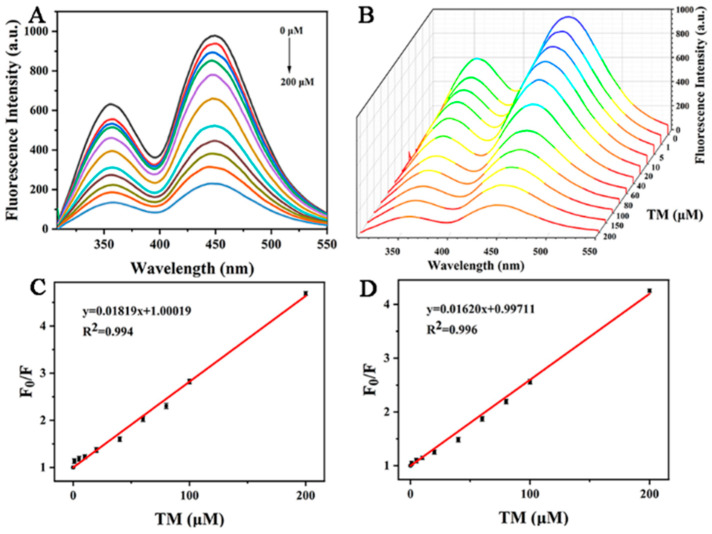
(**A**) Fluorescence emission spectra of Cu-CDs in the presence of TM, different colored lines from top to bottom represent 0–200 μM TM concentrations; (**B**) three-dimensional fluorescence emission spectra with the fluorescence quenching degree at 375 nm (**C**) and 450 nm (**D**).

In order to prove its potential analytical application value, 0–200 μM TM was added into the buffer solution of copper-doped quantum dots for quantitative detection. As shown in Figure 8A, the fluorescence quenching degree of Cu-CDs’ double emission peak was inversely proportional to its concentration in the range of 0–200 μM with increasing TM concentration. Figure 8B is the three-dimensional fluorescence spectrum of Cu-CDs with different TM concentrations. Figure 8C is the linear fitting of the fluorescence emission peak of Cu-CDs at 375 nm (peak A). The obtained calibration equation was F_0_/F = 1.00019 + 0.01819 [TM], correlation coefficient R^2^ = 0.994, where F and F_0_ represent the presence and absence of TM, respectively. The fluorescence emission intensity of Cu-CDs solution was calculated; the detection limit was 0.75 μM, and the limit of quantitation was 2.25 μM. Figure 8D is a linear fitting of the fluorescence emission peak of Cu-CDs at 450 nm (peak B). The calibration equation was F_0_/F = 0.99711 + 0.01620 [TM]. The correlation coefficient R^2^ was 0.996, the detection limit was calculated as 0.78 μM and the limit of quantitation was 2.34 μM, which was compared with other strategies reported in the literature as summarized (Table 1).

### 3.7. Test of Thiophanate Methyl in Actual Samples

**Figure 9:**
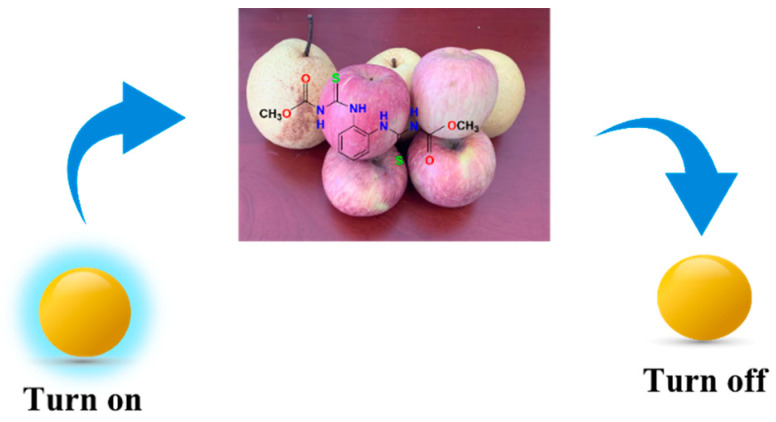
Schematic diagram of pesticide residue detection in pear and apple.

The Cu-CDs sensor was applied to detect fruit (pear and apple) to test the applicability and accuracy of the prepared Cu-CDs sensor in actual samples (Figure 9). After adding a certain concentration of TM into the solution of the sample, fluorescence analysis was carried out. As shown in Table 2, we tested three pear samples and three apple samples, and the recoveries of pear and apple samples were 79.70–91.15% and 81.20–93.55%, respectively. The relative standard deviations (RSD) were less than 4.23% and 3.78% for pear and apple samples, respectively. The results showed that the sensor is feasible and reliable in real-sample TM detection.

## 4. Conclusions

Cu-CDs with dual-emission fluorescence characteristics were prepared by introducing transition metal doping for the rapid detection of pesticide residues in food. Under the excitation of a single excitation wave of 290 nm, Cu-CDs showed two obvious fluorescence emission peaks at 375 nm and 450 nm. Our detailed study showed that the introduced transition metal copper could not only enhance the fluorescence intensity of CDs but also provide abundant surface-active sites. A fast and sensitive sensor was designed for the determination of TM residues in agricultural products based on the principle that TM could chelate with the active site on the surface of Cu-CDs and have π-π stacking with its benzene ring, which caused the synchronous fluorescence quenching of Cu-CDs. The fluorescence quenching can be observed immediately. By optimizing the detection conditions, the fluorescence quenching degree of Cu-CDs showed a good linear relationship with TM concentration. In fruit samples, the low detection limits for the pear and apple were 0.75 μM and 0.78 μM, respectively, and the limits of quantitation for the pear and apple were 2.25 μM and 2.34 μM, respectively. Our results show that the product has high sensitivity, which can be verified in the detection of agricultural product samples.

## Figures and Tables

**Table 1:** Results of our work are compared with the previous TM-detection methods.

Name of the Probe	Detection Range (μM)	LOD (μM)	Reference
LC-MS/MS	0.23–0.53	61.3	[47]
SPE-HPL	-	43.8 × 10^3^	[48]
AuNRs	-	14.6	[49]
Surface-enhanced Raman	0.00–23.4	-	[50]
Cu-CDs	0.00–0.65	0.75 (Peak A) 0.78 (Peak B)	This work

“-” means not mentioned.

**Table 2:** Determination of TM in real samples (*n* = 3).

Sample	Added TM (μM)	Determined TM (μM)	Recovery (%)	RSD (%)
	10.00	8.97	79.70	3.76
Pear	20.00	18.23	91.15	3.59
	40.00	35.20	82.38	4.23
Apple	10.00	8.87	81.20	3.46
	20.00	17.51	93.55	2.68
	40.00	37.42	87.53	3.78

## Data Availability

The dates are available from the corresponding author.

## Supplementary Materials

The following supporting information can be downloaded at: https://www.mdpi.com/article/10.3390/foods11213336/s1, Figure S1: Fluorescence emission spectra of Cu-CDs with different reaction ratios (A), different reaction temperatures (B) and different reaction times (C); Figure S2: (A) pH, (B) temperature and (C) reaction time of TM effect on fluorescence intensity of Cu CDs.
