# Supplementary material for: Copper-Modified Double-Emission Carbon Dots for Rapid Detection of Thiophanate Methyl in Food
Source: Foods. 2022 Oct 24;11(21):3336. doi: 10.3390/foods11213336 (PMC9656121; doi:10.3390/foods11213336)
Supplement: Supplementary file 1 [file foods-11-03336-s001.zip › foods-1888046-supplementary.pdf]

Synthesis of Copper Modified Double Emission Carbon Dots and Their Application in Rapid Detection of Thiophanate Methyl

Rongrong Gu[1], Chunna Zhu[1], Xiaona Yue[1], Juan Hu, Yang Xu, Sheng Ye*and Jing Zhu*

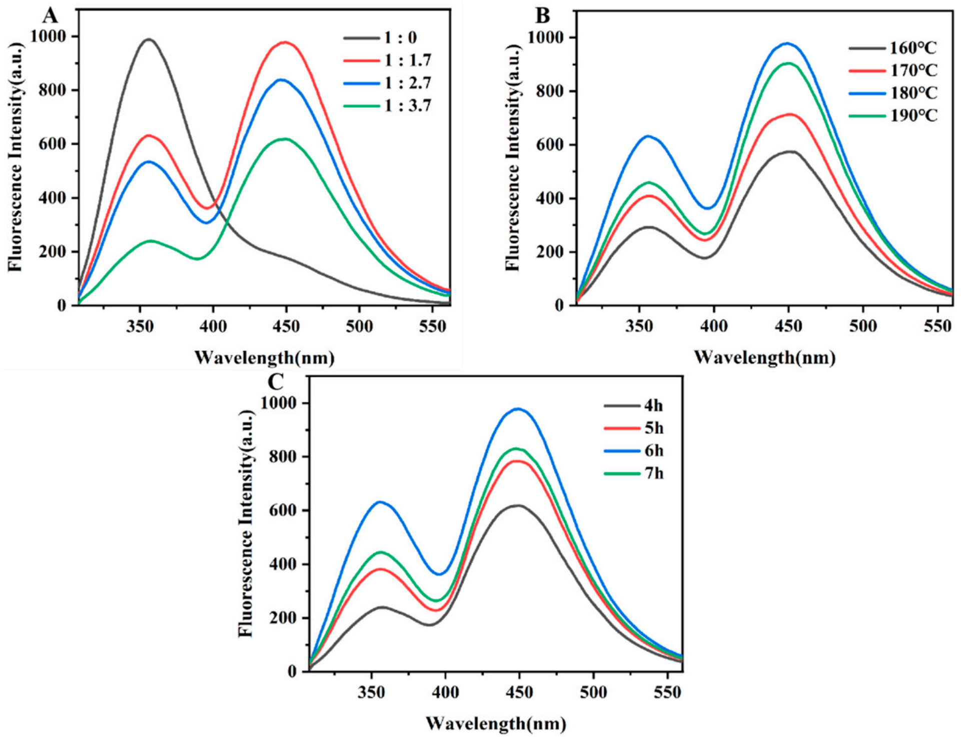

Figure S1 Fluorescence emission spectra of Cu-CDs with different reaction ratios (A), different reaction temperatures (B) and different reaction times (C).

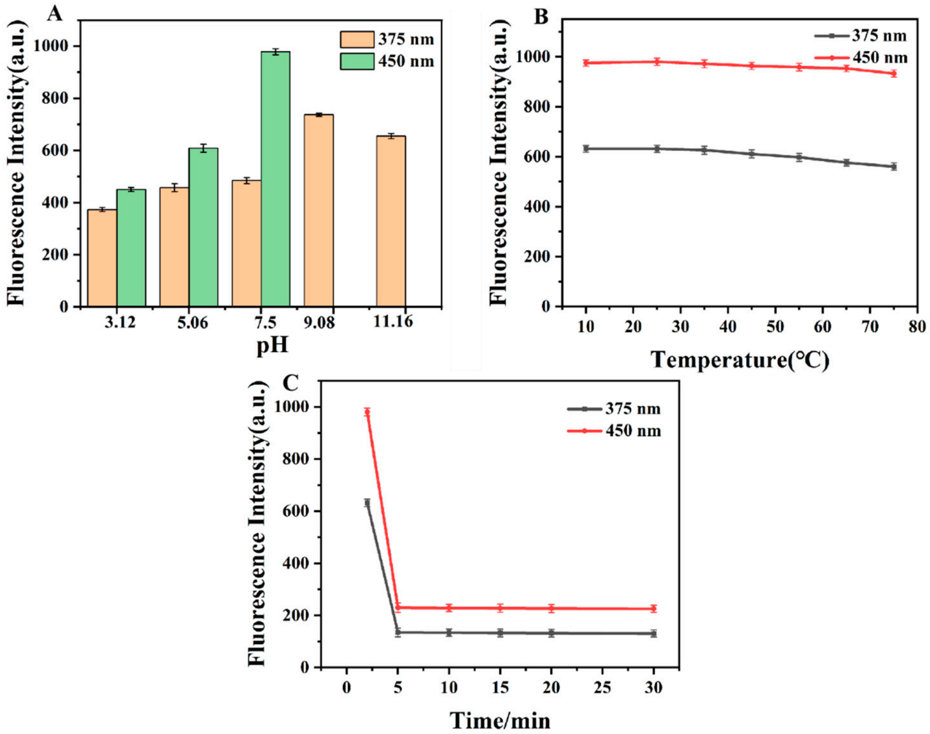

Figure S2. (A) pH, (B) temperature and (C) reaction time of TM effect on fluorescence intensity of Cu CDs.
